# Supplementary material for: Comparison of Ruptured Intracranial Aneurysms Identification Using Different Machine Learning Algorithms and Radiomics
Source: Diagnostics (Basel). 2023 Aug 9;13(16):2627. doi: 10.3390/diagnostics13162627 (PMC10453422; doi:10.3390/diagnostics13162627)
Supplement: Supplementary file 1 [file diagnostics-13-02627-s001.zip › diagnostics-2521228-supplementary.pdf]

Supplementary Materials

Inter-observer and intra-observer reproducibility analysis

Inter-observer and intra-observer reproducibility analysis was performed on 50 patients randomly selected from center 1. The volume of interest (VOI) of each patient was semi-automatically segmented again by the same radiologist after the interval of 3 days and by another radiologist with the same method. After the extraction of radiomics features, the interclass correlation coefficient (ICC) was calculated to assess the reproducibility of radiomics features. The results showed that 99 (90.8%) radiomics features showed ICC > 0.8 in the inter-observer reproducibility analysis and 101 (92.7%) radiomics features showed ICC > 0.8 in the intra-observer reproducibility analysis.

Harmonization of Radiomics Features

After radiomics features extraction, harmonization in the feature domain was performed. First, variables with zero variance were excluded from analyses, and the missing values were replaced by the median. Then the data were standardized by the standardization.

Definition of radiomics features

The Skewness measures the asymmetry of the distribution of values about the Mean value. Depending on where the tail is elongated and the mass of the distribution is concentrated, this value can be positive or negative. The Elongation shows the relationship between the two largest principal components in the ROI shape. For computational reasons, this feature is defined as the inverse of true elongation. The values range between 1 (where the cross section through the first and second largest

principal moments is circle-like (non-elongated)) and 0 (where the object is a maximally elongated: i.e. a 1 dimensional line). The Maximum means the maximum gray level intensity within the ROI. The Cluster Shade is a measure of the skewness and uniformity of the GLCM. A higher cluster shade implies greater asymmetry about the mean. The Zone Percentage measures the coarseness of the texture by taking the ratio of number of zones and number of voxels in the ROI. And the Strength is a measure of the primitives in an image. Its value is high when the primitives are easily defined and visible, i.e. an image with slow change in intensity but larger coarse differences in gray level intensities.

A brief description of machine learning algorithms we adopted

1. Support Vector Machine (SVM): SVM is a well-established algorithm for binary classification tasks. It works well with high-dimensional data like radiomics features and can handle non-linear relationships effectively.

2. Decision Tree: Decision trees are intuitive and easy to interpret, making them suitable for gaining insights into feature importance. Additionally, they are capable of handling non-linear relationships and interactions between features.

3. XGBoost (Extreme Gradient Boosting): XGBoost is a widely used ensemble learning technique known for its high predictive accuracy and ability to handle complex data relationships. It's especially effective for structured data like radiomics features.

4. Gaussian Naive Bayes (GNB): GNB is a probabilistic algorithm that performs well with small datasets and is particularly useful for problems with high-dimensional feature spaces.

5. Logistic Regression: Logistic regression is a common choice for binary classification tasks and serves as a baseline model due to its simplicity and interpretability.

6. Random Forest: Random forests are robust ensemble models that can capture complex interactions and reduce overfitting. They are suitable for high-dimensional feature sets.

7. k-Nearest Neighbors (KNN): KNN is a non-parametric algorithm that can be effective when there is a local structure in the data, making it useful for some medical imaging tasks.

8. Bagging Classifier: Bagging combines multiple models to reduce variance and improve generalization, making it suitable for enhancing the stability of decision trees.

9. AdaBoost: AdaBoost is an adaptive boosting algorithm that focuses on misclassified data points, which can be advantageous in improving the performance of weak learners like decision trees.

10. Gradient Boosting: Gradient boosting builds decision trees sequentially, each correcting the errors of its predecessor, making it powerful for capturing complex patterns in the data.

11. LightGBM (LGBM): LGBM is an efficient gradient boosting framework known for its high performance and speed, making it well-suited for large-scale datasets.

12. CatBoost: CatBoost is another gradient boosting algorithm that handles categorical features well and requires minimal data preprocessing.

Table S1. Characteristics of patients with intracranial aneurysm in the training and validation cohorts

Variables	Training cohort (n = 403)	Validation cohort (n = 173)	*P* value
Age, y, median [IQR]	63.0 [53.0, 71.0]	62.0 [52.0, 71.0]	0.694
Gender			0.645
Male, n (%)	165 (40.9)	75 (43.4)	
Female, n (%)	238 (59.1)	98 (56.5)	
Hypertension history, n (%)	243 (60.3)	103 (59.5)	0.926
aSAH history, n (%)	1 (0.2)	0 (0)	1.000
Aneurysm size, mm, median [IQR]	3.2 [2.2, 4.8]	3.3 [2.4, 5.1]	0.318
PHASES, median [IQR]	2.0 [1.0, 4.0]	2.0 [1.0, 5.0]	0.436
Location			0.810
ICA, n (%)	280 (69.5)	119 (68.8)	
ACA/ACOM, n (%)	48 (11.9)	24 (13.9)	
MCA, n (%)	48 (11.9)	17 (9.8)	
PCOM/Posterior circulation, n (%)	27 (6.7)	13 (7.5)	

Data are noted as mean and standard deviation, median and interquartile ranges or numbers and percentages in parenthesis. SD, standard deviation; aSAH, aneurysmal subarachnoid hemorrhage; ICA, internal carotid artery; ACA, Anterior cerebral artery; MCA, Middle cerebral artery; ACOM, anterior communicating artery; PCOM, posterior communicating artery.

Table S2. The correlations of between the lesion volume and the seven radiomics features that we introduced into machine learning analysis.

Spearman Correlation		original_firstorder_Maximum	original_firstorder_Skewness	original_glcm_ClusterShade	original_gldm_DependenceEntropy	original_glszm_ZonePercentage	original_ngtdm_Strength	original_shape_Elongation
original_shape_VoxelVolume	r	0.043	0.198**	-0.497**	-0.624**	0.983**	-0.758**	-0.426**
	p value	0.308	<0.001	<0.001	<0.001	<0.001	<0.001	<0.001

Table S3. The results of Delong test across 12 machine learning algorithms.

Models		RF	SVM	DT	XGB	GNB	LR	KNN	BC	AdaBoost	GB	LGBM	CatBoost
RF	Z score	N/A	-1.2405	-2.9777	-0.6513	-0.8865	-0.6158	-1.8989	-0.3735	2.8926	4.1510	0.2977	2.5722
	p	N/A	0.2148	0.0029	0.5148	0.3754	0.5380	0.0576	0.7088	0.0038	<0.0001	0.7659	0.0101
SVM	Z score	-1.2405	N/A	-1.6402	0.9362	0.3950	0.9798	-1.0286	1.1441	3.4241	3.6297	1.6870	3.8472
	p	0.2148	N/A	0.1010	0.3492	0.6929	0.3272	0.3037	0.2526	0.0006	0.0003	0.0916	0.0001
DT	Z score	-2.9777	-1.6402	N/A	2.6398	1.8507	2.0267	0.8395	3.0700	4.8704	4.8654	2.9567	4.1165
	p	0.0029	0.1010	N/A	0.0083	0.0642	0.0427	0.4012	0.0021	<0.0001	<0.0001	0.0031	<0.0001
XGB	Z score	-0.6513	0.9362	2.6398	N/A	-0.5092	-0.2413	-1.6637	0.2833	3.1625	4.5699	1.0154	3.1630
	p	0.5148	0.3492	0.0083	N/A	0.6106	0.8093	0.0962	0.7770	0.0016	<0.0001	0.3099	0.0016
GNB	Z score	-0.8865	0.3950	1.8507	-0.5092	N/A	0.5254	-1.3084	0.6996	3.2812	3.3132	1.1532	3.3287
	p	0.3754	0.6929	0.0642	0.6106	N/A	0.5993	0.1907	0.4842	0.0010	0.0009	0.2488	0.0009

LR	Z score	-0.6158	0.9798	2.0267	-0.2413	0.5254	N/A	-1.5049	0.4300	3.1052	3.1920	0.9177	3.3201
	p	0.5380	0.3272	0.0427	0.8093	0.5993	N/A	0.1324	0.6672	0.0019	0.0014	0.3588	0.0009
KNN	Z score	-1.8989	-1.0286	0.8395	-1.6637	-1.3084	-1.5049	N/A	1.9415	4.0109	3.9352	2.2890	3.9674
	p	0.0576	0.3037	0.4012	0.0962	0.1907	0.1324	N/A	0.0522	0.0001	0.0001	0.0221	0.0001
BC	Z score	-0.3735	1.1441	3.0700	0.2833	0.6996	0.4300	1.9415	N/A	3.0112	4.5211	0.7905	3.4924
	p	0.7088	0.2526	0.0021	0.7770	0.4842	0.6672	0.0522	N/A	0.0026	<0.0001	0.4292	0.0005
ADA	Z score	2.8926	3.4241	4.8704	3.1625	3.2812	3.1052	4.0109	3.0112	N/A	-0.4099	-2.6244	-1.3121
	p	0.0038	0.0006	<0.0001	0.0016	0.0010	0.0019	0.0001	0.0026	N/A	0.6819	0.0087	0.1895
GB	Z score	4.1510	3.6297	4.8654	4.5699	3.3132	3.1920	3.9352	4.5211	-0.4099	N/A	-3.7544	-1.5073
	p	<0.0001	0.0003	<0.0001	<0.0001	0.0009	0.0014	0.0001	<0.0001	0.6819	N/A	0.0002	0.1317
LGBM	Z score	0.2977	1.6870	2.9567	1.0154	1.1532	0.9177	2.2890	0.7905	-2.6244	-3.7544	N/A	2.8465
	p	0.7659	0.0916	0.0031	0.3099	0.2488	0.3588	0.0221	0.4292	0.0087	0.0002	N/A	0.0044

CB	Z score	2.5722	3.8472	4.1165	3.1630	3.3287	3.3201	3.9674	3.4924	-1.3121	-1.5073	2.8465	N/A
	p	0.0101	0.0001	<0.0001	0.0016	0.0009	0.0009	0.0001	0.0005	0.1895	0.1317	0.0044	N/A

Abbreviations: RF, Random forest; SVM, support machine learning; DT, decision-making tree; XGB, eXtreme Gradient Boosting; GNB, Gaussian Naive Bayes; LR, Logistic regression; KNN, k-Nearest Neighbor; BC, Bagging Classifier; ADA, Adaboost; GB, Gradient boosting; LGBM, Light Gradient Boosting Machine; CB, Catboost.
